# Hepatitis B infection and immunity in migrant children and pregnant persons in Europe: a systematic review and meta-analysis

**DOI:** 10.1093/jtm/taae094

**Published:** 2024-07-11

**Authors:** Carla Hobart, Julia M Pescarini, Laith Evans, Haleema S Adil, Shehzhore T Adil, Anna Deal, Jessica Carter, Philippa C Matthews, Sally Hargreaves, Nuria Sanchez Clemente

**Affiliations:** Faculty of Epidemiology and Population Health, London School of Hygiene & Tropical Medicine, Keppel Street, WC1E 7HT, London, UK; Faculty of Epidemiology and Population Health, London School of Hygiene & Tropical Medicine, Keppel Street, WC1E 7HT, London, UK; Centre of Data and Knowledge Integration for Health (CIDACS), Gonçalo Moniz Institute, Oswaldo Cruz Foundation, Rua Waldemar Falcão, 121, Candeal - Salvador/BA CEP: 40296-710, Bahia, Brazil; Centre for Neonatal and Paediatric Infection, St. George’s University of London, Cranmer Terrace, London SW17 0RE, UK; University College London Medical School, 74 Huntley St, London WC1E 6DE, UK; University College London Medical School, 74 Huntley St, London WC1E 6DE, UK; Migrant Health Research Group, Institute for Infection and Immunity, St. George’s University London, Cranmer Terrace, London SW17 0RE, UK; Migrant Health Research Group, Institute for Infection and Immunity, St. George’s University London, Cranmer Terrace, London SW17 0RE, UK; The Francis Crick Institute, HBV Elimination Laboratory, 1 Midland Road, London NW1 1AT, UK; Division of Infection and Immunity, University College London, Gower Street, London WC1E 6BT, UK; Department of Infectious Diseases, University College London Hospital, Euston Road, London NW1 2BU, UK; Migrant Health Research Group, Institute for Infection and Immunity, St. George’s University London, Cranmer Terrace, London SW17 0RE, UK; Faculty of Epidemiology and Population Health, London School of Hygiene & Tropical Medicine, Keppel Street, WC1E 7HT, London, UK; Centre for Neonatal and Paediatric Infection, St. George’s University of London, Cranmer Terrace, London SW17 0RE, UK; Migrant Health Research Group, Institute for Infection and Immunity, St. George’s University London, Cranmer Terrace, London SW17 0RE, UK

**Keywords:** Migrants, chronic hepatitis B, pregnant, hepatitis, preventions, prevalences

## Abstract

**Background:**

The WHO’s global hepatitis strategy aims to achieve viral hepatitis elimination by 2030. Migrant children and pregnant persons represent an important target group for prevention strategies. However, evidence on the burden of chronic hepatitis B (CHB) infection and the factors affecting its incidence is lacking.

**Methods:**

EMBASE, Global Health, Global Index Medicus, Web of Science and Medline were searched for articles in any language from 1 January 2012 to 8 June 2022. Studies reporting CHB prevalence, disease severity, complications and/or prevention strategies, including vaccination, prevention of vertical transmission and access to care/treatment for migrant children and pregnant migrants, were included. Pooled estimates of CHB prevalence and hepatitis B vaccination (HBV) coverage among migrant children were calculated using random effects meta-analysis.

**Findings:**

42 studies were included, 27 relating to migrant children and 15 to pregnant migrants across 12 European countries, involving data from 64 773 migrants. Migrants had a higher incidence of CHB than host populations. Among children, the pooled prevalence of CHB was higher for unaccompanied minors (UAM) (5%, [95% CI: 3–7%]) compared to other child migrants, including internationally adopted children (IAC) and refugees (1%, [95% CI: 1–2%]). Region of origin was identified as a risk factor for CHB, with children from Africa and pregnant migrants from Africa, Eastern Europe and China at the highest risk. Pooled estimates of HBV vaccine coverage were lower among UAM (12%, [95% CI: 3–21%]) compared to other child migrants (50%, [95% CI: 37–63%]).

**Conclusion:**

A range of modifiable determinants of HBV prevalence in migrant children and pregnant persons were identified, including sub-optimal screening, prevention and continuum of care. There is a need to develop evidence-based approaches in hepatitis care for these groups, thereby contributing towards global viral hepatitis elimination goals.

## Introduction

Chronic hepatitis B (CHB) is defined as the persistence of hepatitis B surface antigen (HBsAg) for 6 months or more after acute infection with hepatitis B virus (HBV).^1^ There are an estimated 296 million people living with CHB globally,^2^ including 6 million children younger than 5 years.^3^ The majority live in highly endemic areas (where the prevalence of CHB is >8% of the population), including the East Asian, Eastern Mediterranean, Western Pacific and African regions.^4^ In these areas, most infections occur during infancy or childhood due to vertical transmission (mostly during childbirth but occasionally during pregnancy)^5^ or due to horizontal household transmission between children.^6^ The risk of progression from acute to chronic infection is higher when infection occurs in younger age groups, compared to populations who are infected later in life. Progression rates can reach up to 90% when infection occurs in the neonatal period, compared to 20% in childhood, and <5% in immunocompetent adults.^7–9^ In 2019, 820 000 deaths were attributable to CHB globally, many of which were secondary to cirrhosis and hepatocellular carcinoma (HCC).^10^

The sustainable development goals highlighted the importance of combatting hepatitis, and in 2016, the WHO launched a global strategy for achieving viral hepatitis elimination as a public health threat by 2030,^2^ defined as a 90% reduction in incidence and a 65% reduction in mortality compared with the 2015 baseline.^2^ Paediatric and vaccine-specific targets included 90% coverage of the 3-dose vaccine regimen and reducing HBsAg in children under 5 years to <0.1%.^11^ It also forms part of the triple elimination initiative, which aims to synergise efforts to prevent mother-to-child transmission of HIV, HBV and syphilis.^12^ The success of achieving these targets is highly reliant on preventative interventions delivered during pregnancy and childhood, such as antiviral (tenofovir) prophylaxis from 28 weeks gestation in women with high viral loads (≥200 000 IU/ml),^13^ a timely (within 24 hours) birth dose of hepatitis B vaccination, followed by 2–6 doses in infancy, and HBV immunoglobulin (HBIG) administration at birth in high-risk cases.^14–16^ Global coverage with three doses of the hepatitis B vaccine is estimated to be at 84%,^17^ with a target rate of 90% by 2030.^10^

Progress towards this fast-approaching WHO global deadline has been unequal across high burden populations. In Europe, a region with low endemicity that has seen recent migration waves from high (>8%) and intermediate (2–7%) CHB endemic regions,^4^ migrant populations are at higher risk of CHB than the native-born population,^18^^,^^19^ and migrants from countries where CHB is highly prevalent (≥2%) account for 25% of all CHB infections in the EU.^20^ Migrant children and pregnant persons in Europe are thus an important area of focus for regional prevention and care programmes.^19^ However, vaccination and screening practices vary across the region, and migrant populations are often overlooked by health systems and subject to inequities in accessing healthcare (including vital preventative screening and vaccination programmes) in host countries.^21–23^ This has resulted in migrants being under-immunised for hepatitis B and other communicable diseases.^21^ Although all 31 EU/EEA countries recommend vaccination for children in high-risk groups, and 27 even recommend universal childhood HBV vaccination, only seven EU/EEA countries have a national policy for screening migrants specifically for HBV.^24^ With regards to antenatal screening for HBV, universal screening is national policy in seven EU/EAA countries, and opt-out screening is policy in a further 14.^25^

Migrant children and pregnant persons are not only a key at risk group for CHB but are also cared for within distinct healthcare pathways and services (i.e. antenatal care, paediatric clinics and childhood vaccination services) within which HBV prevention and treatment could potentially be more easily administered. This includes opportunities to deliver prevention, most notably HBV vaccination, opportunistically if flexibility exists around the commissioning of these services. A strong evidence base is therefore vital for curating informed and tailored interventions for the prevention of CHB in these specific vulnerable groups. While evidence on the prevalence of CHB in migrants has previously been the subject of systematic reviews, there is currently no evidence synthesis available on CHB in migrant children and pregnant persons specifically.^26^^,^^27^

## Methods

We conducted a systematic review and meta-analysis, according to PRISMA guidelines,^28^ to determine CHB prevalence, disease severity and complications and factors affecting incidence, including primary and secondary prevention strategies (vaccination, prevention of vertical transmission, screening and access to care/treatment, respectively) among migrant children and pregnant migrant populations in Europe.

### Inclusion and exclusion criteria

The inclusion and exclusion criteria were developed using the Joanna Briggs Institute methodology^29^ (Appendix Table AA1, p. 1). We included primary research studies with data on CHB prevalence (primary outcome) and disease severity/complications and factors affecting CHB incidence, including vaccination status, prevention of vertical transmission, screening and access to care/treatment (secondary outcomes), among migrant pregnant women and/or children aged 18 years or less born outside the country of study and living in the EU, EEA, UK and Switzerland (i.e. first generation migrants) in all migrant groups (Appendix Box 1, p. 2). Observational studies, case reports and systematic reviews were included, and comments/editorials, conference abstracts and modelling studies were excluded. Studies published before 2012 were excluded to ensure that the evidence obtained was consistent with current migration trends and CHB prevention approaches, such as global vaccination recommendations.

### Search strategy and study selection

We searched EMBASE, Global Health, Global Index Medicus, Web of Science and Medline for primary research articles in any language published from 1 January 2012 to 8 June 2022 combining English-language keyword search terms and medical subject headings using Boolean operators relating to migrants, prevalence, Hepatitis B and Europe (Appendix Figure AA1, pp. 2–5). Grey literature of relevant governmental and non-governmental organisations [e.g. WHO, European Centre for Communicable Disease Control (ECDC)] was consulted. Bibliographies of the included studies were hand-searched for additional relevant studies.

Google was searched with key terms (including ‘migrant’, ‘Hepatitis B’ and ‘Europe’) and the first 50 results were reviewed. No language restrictions were applied, and Google translate and DeepL translator were used as required.^30^^,^^31^

### Data screening, extraction and synthesis

Records were imported into EndNote and duplicates were deleted. Title, abstract and full-text screening were carried out according to the aforementioned inclusion and exclusion criteria. Data were extracted and tabulated separately for migrant children and pregnant women. Using Microsoft Excel, a standardised form was developed to extract data on the following: author and year of publication of study, study setting and location, study design, number of participants (where relevant), refugee and migrant demographics (country of origin, legal status), age group and gender (for children), CHB prevalence, disease severity or complications, HBV vaccination status/coverage, screening and access to CHB care services.

To estimate the pooled CHB prevalence and pooled HBV vaccine coverage among migrant children, we conducted meta-analyses using the random effects model available from Stata 17 metaprop function.^32^ This enabled the calculation of 95% confidence intervals using the statistical score and the exact binomial method, and it incorporated the Freeman–Tukey arcsine double proportions transformation.^33^ This method also models intra-study variability using the binomial distribution. Inter-study heterogeneity was described using the I2 statistic, which describes the percentage of variation across studies that is due to heterogeneity rather than chance. The weights of the studies were also calculated to account for the differing sample sizes of the studies. We calculated separate pooled estimates for unaccompanied minors (UAM) and children with other migration statuses, including those described as migrants, refugees, asylum seekers and international adopted children (IAC). We excluded studies where sample sizes were not specified or where prevalence data for UAM and other migration types were not disaggregated. Two studies that included data on young people aged 0–20 and 13–25 years were included.^34^^,^^35^

The risk of bias was assessed for all studies was carried out using the Newcastle–Ottawa scoring (NOS) system (score out of 9) and an adapted scoring system for cross-sectional studies (score out of 8). Studies that scored a total of 8 or 9 points were considered to have a low risk of bias; 7 or 6 points were considered to have a medium risk of bias; and 5 points or less were considered to have a high risk of bias.

## Results

### Summary of included studies

**Figure 1:**
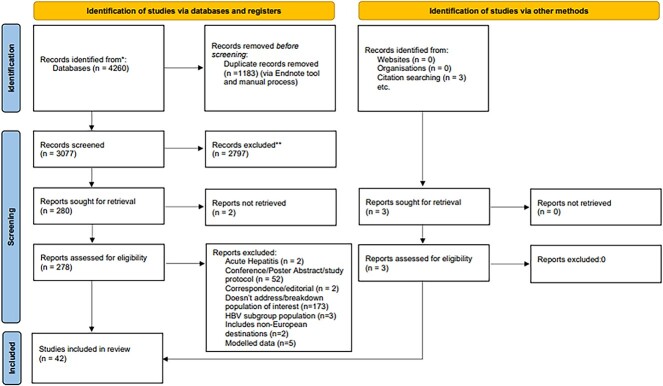
PRISMA study selection flow diagram. Adapted from the PRISMA 2020 Statement^28^

**Figure 2:**
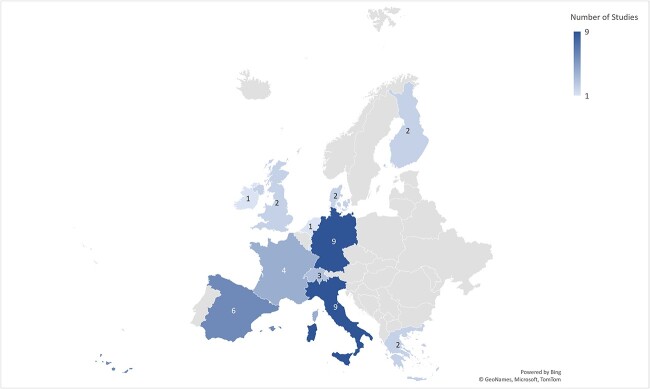
Number of studies by migrant destination. (N.B.: one study from Luxembourg not visible)

Our search returned 4260 articles, of which 3077 were screened (*n* = 278 full text), 42 included in the final study (Figure 1), and 23 in the meta-analyses. The studies reported on CHB prevalence (*n* = 35) in migrant children (*n* = 23) and pregnant women (*n* = 12), disease severity (*n* = 5), HBV vaccination/susceptible population (*n* = 26), screening (*n* = 5) and specialised migrant care pathways (*n* = 5), in a total of 64 773 migrants (Tables 1 and 2). The studies reported cases from 10 different countries (Figure 2), including Italy (*n* = 9), Germany (*n* = 8) Spain (*n* = 6), France (*n* = 4), Denmark (*n* = 2), United Kingdom (*n* = 2), Finland (*n* = 2), Ireland (*n* = 1), The Netherlands (*n* = 1) and Greece (*n* = 1). 

**Table 1:** Details of all paediatric migrant hepatitis B studies

Study	Year	Country	Study Setting	Study Purpose	Study Type	Study Population & country/region of origin	Inclusion Age Range (years)	How identified	Sample size	Prevalence Chronic HBV (%)	Vaccination Coverage (%)	Susceptible (%)	Age of Sample/at diagnosis^a^	Gender Split of HBV + ve (%male)	Gender Split of Sample (% male)	Screening/care pathway information	Data on complications, late severe disease
Belhassen-Garcia *et al*.,	2015	Spain	Salamanca	Assess imported transmissible diseases in migrants from low income areas	Cross-sectional (medical records and physical exam)	Immigrant children and adolescents from E/W/S/C Africa, North Africa and Latin America	0–18	Screening in immigrants < 18 yrs	350	4.3	39.7	32	15	86.7	53.4		7/15 patients with CHB had detectable HBV viral loads
Bergevin *et al*.,	2021	France	Dedicated migrant medical consultation service in 1 hospital, Paris	Describe health issues of newly arrived UAMs managed in a dedicated paediatric consultation service	Cross-sectional (single-centre retrospective)	UAM from Africa or Afghanistan	0–18	Visiting dedicated consultation service	90	8.0					89.0	Average time to 1^st^ consult =3 months. UMs have free healthcare until adulthood	
Finnegan *et al*.,	2015	Ireland	Specialist clinic for children born to HBV infected mothers	Describe long-term follow up of children born to HBV infected mothers	Cross-sectional (retrospective)	Children with chronic HBV infection attending specialist clinic (from Africa, Central Asia, Eastern Europe)	0–18	Children born to HBV infected mothers are referred	57				6 m-17 yr	60	60	69% born in Ireland received adequate prophyl. 0% born outside received adequate prophy l. 19% received vaccine alone.	7% (n = 4) developed complication of CHB. n = 3 had fibrosis n = 3 received antivirals
Fougere *et al*.,	2018	Switzerland	Hospital reference centre for the children of asylum seekers in region. More than 90% of migrant children in Lausanne/surroundings followed in this setting.	Estimate HB vaccine protection (using post booster serology). Those with anti-HBs ≥ 100 post booster were assumed to be vaccinated.	Prospective single-centre cohort	Migrant children from Eritrea, Iraq, Syria	1–18	Research study	200	<1%	81		9 yr*/ 12 yr -one case		51	Migrants should have 2 visits (for first dose of vaccine and serology 4–6 weeks later)	
Genton *et al*.,	2019	Switzerland	Migrant care facility clinic in canton of Vaud (service for asylum seekers)—but data across multiple healthcare structures primarily used by this cohort	Describe the overall clinical profile and the care pathways of unaccompanied minor asylum seekers	Cross-Sectional (retrospective study, information extracted from medical records)	UAM (asylum seekers). Afghanistan, Eritrea, Somalia, Syria	0–18	Routine screening of newly arrived children.	109	2.8					87.2	Screening of newly arrived UAMs takes place	
Giordano *et al*.,	2018	Italy	University hospital of Palermo, Sicily	Examine immunisation status of IAC and compare it with vaccination certificates, focusing on measles, mumps, rubella (MMR) and hepatitis B (HBV)	Cross-sectional	Internationally adopted children (IAC). Country/region of origin not specified	0–18	Screening of internationally adopted children	79 (for HBV)	0.0	65.8	34	84 mon		62	Concordance between serology and vaccination records is 71%	
Hahne *et al*.,	2012	Netherlands	Population study	Assess differences in prevalence of HBV infection in The Netherlands between 1996 and 2007, and identify risk factors for HBV infection in 2007	Cross-sectional (seroprevalence)	Representative sample of Dutch population (with oversampling or largest migrant groups from Suriname, Turkey, Morocco, Dutch Antilles, Aruba, Indonesia	0–79	Prevalence study (no data if already aware or not prior to study)	2007: 6246 (0–14 = 1476, 14–29 =1002)	2007 1st gen: 0–14: 2.3% 15–29: 22.3% 2nd gen 0–14: 1.7% 15–29: 2.4%							
Hampel *et al*.,	2016	Germany	5 initial refugee reception centres in Northern Germany	Assess prevalence of hepatitis B and the vaccination status of refugee population	Cross-sectional (retrospective descriptive data analysis)	Refugees. Country/region or origin not reported	0–17	Every refugee (newly arrived) who sought medical treatment for acute complaints offered testing	62 (aged under 18 yrs)	1.6	50			0%	80.6	Initial screening is offered but loss to follow-up and lack of joined up care is common	
Hourdet *et al*.,	2020	France	Health care access centre for vulnerable populations, Paris	Assess the health status of this population	Cross-sectional (retrospective, observational, monocentric)	Patients self-reporting as UAM but not recognised as such by the state from Guinea, Ivory Coast, Mali	0–18	Screening offered when visiting a dedicated consultation service	301 -total 1035 consultations	12.8			16		95	Jurisdictional framework around this status unclear. Precarious access to care	
Hübschen *et al*.,	2012	Luxembourg	Unclear	Investigate prevalence of IgG antibodies against different vaccine-preventable diseases in newcomers to Luxembourg	Cross-sectional	Refugees or asylum seekers (newly arrived) from Albania, Montenegro, Bosnia and Herzegovina, Middle East, Asia, Africa, Russia	13–25	Vaccine coverage study	131 (age 13–25)		25					Majority of migrants lacked antibodies to one or more VPDs	
Jablonka *et al*.,	2015	Germany	single reception centre	Determine seroprevalence of antibodies against hepatitis A–E in an unselected cohort of refugees and asylum seekers during Middle East crisis	Cross-sectional	Refugees and asylum seekers from Africa, East MediterraneanEurope, SE Asia	0–17	On arrival screening (mandatory)	91	0.0	40.7	32			74.7		
Janda *et al*.,	2020	Germany	Single paediatric consultation service for UMs, Municipal area Southwest Germany	Understanding frequency and clinical presentation of IDs among minor refugees, evaluate the performance and practicability of screening recommendation	Cross-sectional	UAM (refugee) from Africa (93.6%), Asia and Southern Europe	0–18	On arrival screening	776 with HBV (of 890)	7.7			16*		94	Problems with follow-up and retention in HBV service. Unable to offer antivirals	75% of URMs with active HBV were HBeAg -ve and had low viral loads in blood (<10 IU/ml)
Kloning *et al*.,	2018	Germany	2 paediatric practices and one collective housing for refugees in region of Bavaria, Germany	Investigate the morbidity profile and the sociodemographic characteristics of unaccompanied refugee minors (URM)	Cross-sectional (retrospective data derived from medical data records of routine first medical exam)	UAM (refugee) from Afghanistan, Eritrea, Somalia, Syria	0–18	On arrival screening (mandatory)	113	8	3.2	58	16*		93.5	No standardised pathway. Follow-up challenges due to frequent relocations	
Maasen *et al*.,	2017	Germany	On arrival screening	Describe microbiological screenings for infection control in unaccompanied minor refugees undertaken by the German Armed Forces Medical Service	Cross-sectional	UAM (refugee) from Afghanistan, Algeria, Benin, Egypt, Ghana, Guinea, Iran, Iraq, Libya, Morocco, Palestine, Pakistan	0–18	On arrival screening (mandatory)	190 (from total sample of 219)	1.6			13–18	100	100		
Marquardt *et al*.,	2016	Germany	On arrival screening in a private outpatient clinic for internal and tropical medicine, Bielefeld	Investigate the physical and mental disease burden of unaccompanied asylum-seeking adolescents	Cross-sectional	UAM (asylum-seeking adolescents) from Africa, Asia (mostly Afghanistan, Guinea, Morocco)	12–18	On arrival screening	101 tested for HBV (of 102)	7.9	14.9		16	100	76.5	On arrival screening available	Two children required antivirals
Marrone *et al*.,	2020	Italy	Reception centres, Rome	Address prevalence of infectious diseases in a population of unaccompanied immigrant minors	Cross-sectional	UAM from Africa, SE Asia, Eastern Europe	0–18	On arrival screening	879	2.5	18.2	76	17	100	97.6	On arrival screening available	
Mockenhaupt *et al*.,	2016	Germany	Berlin travel and tropical medicine clinic GeoSentinel site	Present results of screening a cohort of unaccompanied Syrian minors (UAMs)	Cross-sectional	UAM from Syrian	0–18	On arrival screening	488	0.0					94		
Monpierre *et al*.,	2016	France	Regional system for isolated foreign minors in Gironde	Describe data on overall health status obtained from a systematic medical check-up offered to URMs	Cross-sectional (data descriptive)	UAM (refugee) from Africa, Asia, Eastern Europe	0–18	On arrival screening (systematic)	235	6.0			16		89.8	On arrival screening available	
Norman *et al*.,	2021	Spain	Migrant referral centre, Madrid	Describe seroprevalence rates for potentially transmissible viral infections in migrants attended at a referral centre in a major European city	Cross-sectional	Migrants from Africa	0–20	Attended for the first time (symptomatic or asymptomatic referred for a health exam) Unclear if screening was standardised.	96	10.4	24	30					
Olivan-Gonzalvo *et al*.,	2021	Spain	UAM protection centres	Examine the health status and infectious diseases in a cohort of unaccompanied immigrant minors from Africa to Spain	Cross-sectional (retrospective)	UAM (Male, from Africa)	0–18	On arrival screening	622	2.6			16	100	100	Screening on admission to residential care	
Pauti *et al*.,	2016	France	Migrant clinics: Drs of the World Clinics in Lyon, Nice, Paris and Saint-Denis	Present degree of lack of knowledge of the HBV and HCV status of people encountered in 2014, identify socio-demographic factors related to this lack of knowledge. HBV vaccination coverage rate analysed.	Cross-sectional	Persons in precarious conditions for primary health care - 94.5% are migrants from Africa, non-EU European countries, Asia	<15	Attendance at clinic	Unclear		58.1				61.8	Lack of systematic checking of HBV serology/ vaccination status	
Pavlopoulou *et al*.,	2017	Greece	Migrant outpatient clinic of a tertiary Children’s hospital, Athens	Describe demographic, clinical and laboratory characteristics and identify possible determinants among newly arriving immigrant and refugee children	Cross-sectional	Migrant and refugee children mainly from Asia (Afghanistan, Bangladesh), Africa, Europe	0–18	On arrival screening	300	0.0	57.7	42			58.7	Health evaluation for migrant children on arrival and refugees are often referred by NGOs or social services.	
Pohl *et al*.,	2017	Switzerland	Tertiary health care facility in Switzerland in 2015	Describe epidemiology and spectrum of infections of admitted paediatric refugees and asylum seekers	Cross-sectional (retrospective analysis using electronic patient records)	Paediatric refugees and asylum seekers (UAMs = 19.4%) from Africa, Eastern Europe and Asia	0–18	Admitted to hospital	93 patients (105 admissions)				5.7		62	Missed opportunities to offer catch-up vaccinations during admission	
Sollai *et al*.,	2017	Italy	Tertiary health care setting, Italy	Evaluate infectious diseases prevalence in a large cohort of IAC	Cross-sectional	IAC from Africa, Asia and Europe	0–18	On arrival screening	1612	0.8	64.9	35			60	IAC are screened on arrival	
Theuring *et al*.,	2016	Germany	Institute of Tropical Medicine and International Health, Charité- Universitätsmediz Berlin 2014–2015	Screening for infectious diseases among unaccompanied minor refugees	Cross-sectional	UAM (refugees) from Africa, Middle East, Asia, Southern and Eastern Europe	0–18	On arrival screening	1248	1.7			16				
Tiittala *et al*., 2018	2018	Finland	Finland asylum seeker population study	Evaluate public health response to a large influx of asylum seekers to Finland in 2015–2016 re: national guidelines on initial health services and infectious disease screening	Cross-sectional (retrospective register-based)	Asylum Seekers – (accompanied and UAM) from Africa and Asia	0–17	On arrival screening (voluntary)	9031 (3400 of which UAM)	0.8						Screening for Hep B, HIV, syphilis within should occur within 3 m after arrival (but 33% not reached)	
Williams *et al*.,	2020	UK	2 paediatric infectious disease clinics, London	Evaluate a screening programme for infection in UAM children and young people against national guidance and describe rates of identified infection in cohort	Cross-sectional (retrospective, routinely collected healthcare data)	UAM (asylum seeking)	0–18	Voluntary screening (on the basis of an individual risk assessment)	252 attendees, 211 (84%) tested for hepatitis B	4.8			17*		88.6	All UAMs receive a statutory health check and are referred to infectious disease clinic for screening	

a Age is expressed as mean. * = denotes median age.

UAM, unaccompanied migrant.

VPD, vaccine preventable diseases.

**Table 2:** Details of all pregnant migrant hepatitis B studies

Study	Year	Country	Study Setting	Study Purpose	Study Type	Study Population and country/region of origin	How Identified	Sample size	Overall sample prevalence (%)	Migrant prevalence (%)	Native-born prevalence (%)	Proportion Of Infected Women =Migrants	Data on complications, late severe disease	Screening/care pathway information	Transmission
Cochrane *et al*.,	2015	UK	Routine antenatal care, Bristol, UK	Estimate HBV infection prevalence by region of birth in migrant populations in a large city	Cross-sectional (retrospective data linkage)	Pregnant migrant women born in regions with HBV infection prevalence > 2% from all continents.	Routine screening	5840		1.7					
Dalmartello *et al*.,	2019	Italy	Population based survey in Trento Province, Italy	Describe coverage and outcome of screening for rubella, syphilis, toxoplasmosis, CMV, HBV, HCV, HIV, & Group B Streptococcus in pregnancy	Cross-sectional	Pregnant women from No data on countries/ regions of origin.	Routine screening/at delivery	38 712 total women, 9237 migrant (23.8%)	0.9					Foreign citizenship associated with absence of screening	
Dopfer *et al*.,	2018	Germany	3 refugee reception centres in Northern Germany	Assess pregnancy rates and associated primary healthcare needs in three refugee cohorts	Cohort	Refugee women on arrival from Afghanistan, Albania, Azerbajian, Bosnia, Iraq, Montenegro, Nigeria, Syria	Off-site mandatory check-up within their first weeks of residence.	9 pregnant migrants (from 1533 total)		0.0				Variable healthcare provision between centres	
Ehmsen *et al*.,	2014	Denmark	Danish NGO health clinic	Describe characteristics of undocumented migrant patients.	Cross-sectional	Undocumented migrants (including pregnant women) from Global (aggregate data)	Voluntary attendance at NGO clinic relating to pregnancy - then screened	96 pregnant migrants (from 1403 total)		1.0				Median start of ANC = 16 + 4 weeks	
Karatapanis *et al*.,	2012	Greece	1 maternity unit, Athens	Assess seroprevalence of HBV markers among parturient women escaping HBsAg prenatal testing	Cross -sectional (prospective)	Pregnant women from Africa, Albania, Asia, Eastern Europe and Roma	Study	53 HBsAg +ve pregnant migrants (Total 9546 pregnancies)	5.3			77.9 (53/68)		1000 women (10.6%) had no HBsAg status documented. 70.4% of these were immigrants	
Lembo *et al*.,	2017	Italy	Obstetric Division of a Sicilian University Hospital, Southern Italy	Investigate prevalence of HBV and HCV serum markers in a large cohort of pregnant women	Cross-sectional	Pregnant women from Albania, China, Kazakhstan, Morocco, Poland, Romania,	Unclear - in medical records of women delivering	711 pregnant migrants (Total 7558 pregnancies) HBsAg status available for 6128 (81%)	0.5	3.0	0.2				No cases of vertical transmission in babies born to HBsAg +ve mothers. Prophylaxis given to all.
Lo Giudice *et al*.,	2021	Italy	Obstetrics and Gynaecology Operative Unit, Messina	Serological survey on blood samples from pregnant women collected during routine pregnancy screening to evaluate the rate of HBsAg and HCV antibody carriers in a low-endemic territory	Cross-sectional	Pregnant women. No data on countries/ region of origin	Screening	727 pregnant migrants (6169 total pregnancies)	0.4	2.1	0.2	50			
Lopez-Fabal *et al*.,	2013	Spain	Maternity unit in the south of Madrid	Determine prevalence and evolution of markers included in serological screening of pregnant women	Cross-sectional -retrospective	Pregnant women from Africa, Eastern Europe, South-East Asia	Screening	2752 pregnant migrants (8012 total pregnancies) HBV tested in 6939	0.8	1.7	0.4				
Ruffini *et al*.,	2014	Italy	Maternity units of the Marche region, Italy	Evaluate data on congenital and perinatal infections, excluding congenital rubella, in pregnant women in the Marche region (Italy)	Cross-sectional	Pregnant women from Albania, China, Romania, Macedonia, Nigeria, Pakistan, Senegal	Routine screening	397 pregnant migrants (1651 total)	0.8	4.3	0.4	82.5			
Ruffini *et al*.,	2016	Italy	Maternity units of the Marche region, Italy	Evaluate epidemiology of hepatitis B infection in pregnant women, according to country of origin.	Cross-sectional	Pregnant women from Albania, Africa, China, Eastern Europe, Ukraine, Western Pacific	Routine screening	2563 pregnant migrants (10 232 total)	0.8	2.7	0.2			Adherence to screening in migrants: 96.7% (vs 99.4% in native population)	
Ruiz-Extremera *et al*.,	2020	Spain	8 participating hospitals in Spain (Madrid, Seville, Malaga, Oviedo, Almeria, Granada)	Determine prevalence of HBV and HCV in pregnant women in Spain, focusing on country of origin, epidemiological factors and risk of vertical transmission	Multicentre open-cohort study	Pregnant women (Hep B + ve) from Africa, China (and other Asian countries), Eastern Europe, Latin America,	‘routine clinical practice’ - assumed to mean screening but unclear	67 HBV + ve pregnancies	0.4			65.7	14.5% (of all pregnancies) HBeAg +ve 6 women received antivirals during pregnancy (not disaggregated by migrant status).	Immuno-prophylaxis given correctly to all mothers.	Mothers became infected via parenteral (20.9%), sexual (4.5%) and vertical (31.3%) transmission (40.3% unknown)
Sagnelli *et al*.,	2016	Italy	Delivery units at 8 Italian hospitals in different cities in Northern, Central and Southern Italy	Estimate clinical impact of HBV infection in pregnant immigrants and their family members and to identify a clinical approach	Cross-sectional	Pregnant migrant women from Africa, Eastern Europe, East Asia, South America	Unclear - appears routine screening of pregnant women and study screening of family members of HBV + ve	1970 pregnant migrants		7.3			20% were HBeAg +ve. Most HBsAg +ve cases had detectable HBV DNA (50% with VL ≥2000 IU/ml)		
Santiago *et al*.,	2012	Spain	A tertiary hospital in Madrid between August 2007 and October 2008	Determine serological profile of foreign mothers against vertically transmitted infections	Cross-sectional (retrospective)	Pregnant women from Africa, Asia, Europe, Central& South America,	In hospital screening	1214 pregnant migrants women with HBV serology. (Total 2526 migrant and 157 Spanish pregnant women)		2	1.1				
Tasa *et al*.,	2021	Finland	Public maternity clinic and maternity hospital in Helsinki, Finland	Describe the use of maternal health care services and the obstetric outcomes of undocumented women and comparing results with all pregnant women in Finland	Cross-sectional (retrospective register-based)	Undocumented pregnant women from Eastern Europe and Russia, Sub-Saharan Africa, Asia, Middle East	Routine screening	62 undocumented pregnant migrants		3.4	0.2			91% attended antenatal care. 70% <8visits, 40% 0–3 visits. 3 women denied access to care.	
Wendland *et al*.,	2016	Denmark	3 clinics specialising in care for UM (in Copenhagen & Jutland)	Assess screening frequency for HIV, HBV, syphilis in undocumented migrants (UM) and to compare prevalence of infection in UM with DM	Cross-sectional (retrospective)	Undocumented migrant pregnant women from Africa, Eastern Europe, Indian subcontinent Middle East/North Africa Central America, SE Asia	Screening & birth register	219 undocumented pregnant migrants (94 had HBV result)		6.1				Documented migrants have access to screening, undocumented migrants do not and rely on NGOs	

ANC, antenatal care.

Most studies (39/42) were cross-sectional and described the experiences of single centres and therefore consisted of non-representative samples of migrants. Three were cohort studies, one of which undertook representative sampling.^36–38^ All studies reported either on paediatric or pregnant migrants except for one study that reported on pregnant UAM^39^ (Appendix Table A2, p. 5).

Risk of bias scores ranged from 3 to 8, with one study having a low risk of bias, 13 having a medium risk of bias and 28 having a high risk of bias (Appendix Table A3, pp. 6–7).

### Child migrants

#### Prevalence

**Figure 3:**
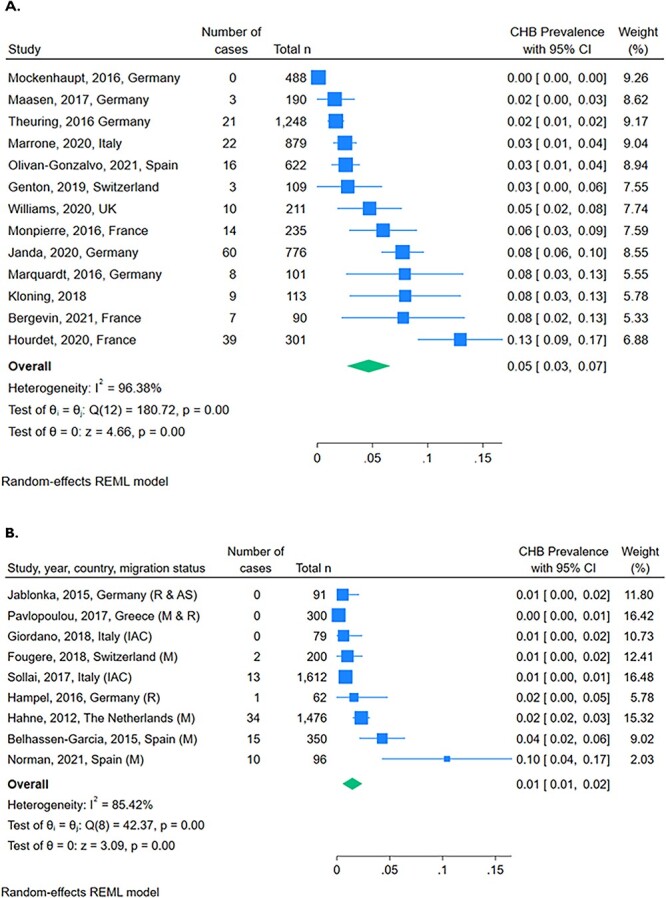
Forest plots of CHB prevalence among migrant children reported by 23 studies. (A) Unaccompanied Migrants (UAM). (B) Other migration status (M, migrants; R, refugees; AS, asylum seekers; IAC, internationally adopted children)

The prevalence of CHB among migrant children reported in 23 studies ranged from 0 to 13% (Table 1 in the article and Appendix Table A4, pp. 7–8). The prevalence varied according to several factors, including migrant type and country of origin. The pooled prevalence of CHB among UAMs was higher (5% [95% CI 3–7]) than among children with other migration statuses, including those described as migrants, refugees, asylum seekers and IAC (2% [95% CI 1–3]) (Figure 3A and B).

One study in France reported a particularly high prevalence of CHB (12.8%) among young people who self-identified as UAM, predominantly from West Africa, but not recognised by the state as such.^40^ By contrast, CHB was rarely identified among the IAC.^41^^,^^42^

In terms of country of origin, a Spanish study looking at prevalence of CHB among 350 migrant children found that all cases were from the WHO Africa Region and that there were no cases among North African and Latin American migrants.^43^ This finding was corroborated in a study describing CHB in UAM in Italy, where children from Gambia and Ivory Coast were found to have a higher prevalence compared to those from Libya.^44^ Paediatric CHB cases were also older (mean age 14.6) compared to vaccinated and non-immune cases (mean ages 11.2 and 11.5, respectively) and more likely to be male in a Spanish study of migrant children from Africa and Latin America and a German sample of UAM seeking asylum.^43^^,^^45^

#### Disease severity and complications

One small Irish study in a clinic for children born to HBV-infected mothers reported complications of CHB—finding fibrosis or inflammation in 7% (4/63) of migrant children living with HBV who originated from Africa, Central Asia and Eastern Europe.^46^ Two studies, one in UAM, reported that the majority (75–93%) of infected children were negative for Hepatitis B e-antigen (HBeAg)—a marker of active HBV replication and high infectivity.^47^^,^^48^ Most also had low viral loads, but the range of viremia differed substantially, and treatment status prior to assessment was not reported.^48^ Antiviral use was reported in studies from Germany in 2/8 adolescents and Ireland, in 3/57 children.^45^^,^^46^

### Factors affecting CHB incidence

#### Vaccination status and susceptible population

Thirteen studies evaluated HBV immunity through vaccination and natural infection using HBV serology, although some of these did not present disaggregated paediatric data (Figure 3).^34^^,^^35^^,^^41–45^^,^^49–54^ Studies that did disaggregate by age group indicated that 32–76% of migrant children are Hepatitis B non-immune (no serological evidence of vaccination or prior infection).^34^^,^^41–44^^,^^49^^,^^54^ Vaccination coverage estimates ranged from 3.2–81% across 13 studies.^34^^,^^35^^,^^41–45^^,^^49–54^

**Figure 4:**
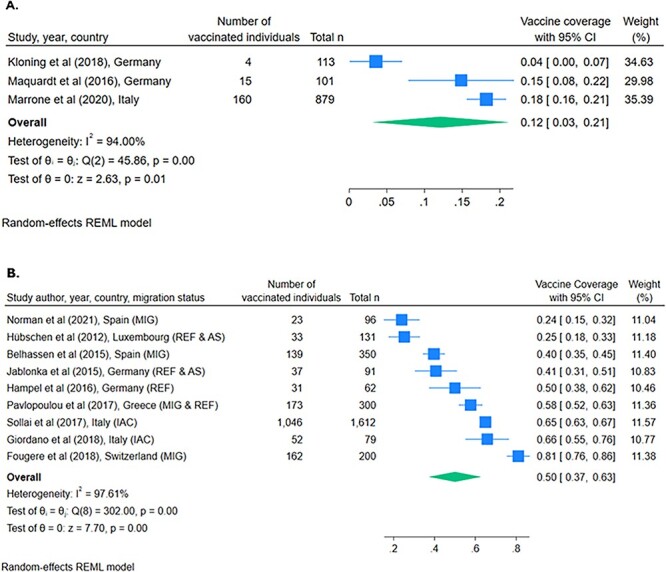
Forest plots of HBV vaccine coverage among migrant children reported by 23 studies. (A) UAM. (B) Other migration status (M, migrants; R, refugees; AS, asylum seekers; IAC, internationally adopted children)

Pooled vaccine coverage estimates were lower among UAMs (12% [95% CI 3–21]) than among other migration categories, including those described as migrants, refugees, asylum seekers and IAC (50% [95% CI 37–63]) (Figure 4A and B).

The association between migrant type and vaccine coverage was also assessed in a Greek study, which found significantly (*P* = 0.015) lower coverage among refugees (defined as asylum seekers and irregular migrant children) compared to immigrants (defined as children of parents with long-term residence permits or those who entered for family reunification).^49^

Vaccination coverage estimates were below coverage rates across most WHO regions in all studies (Figure 2 and Appendix Table A5, pp. 10–11).^35^^,^^52^ Studies also noted that for most children (58.1–79.3%), vaccination records were unavailable, and when available, discrepancies frequently existed between documentation and serological evidence of HBV protection.^41^^,^^42^

#### Screening and care pathways

Eighteen studies from Germany,^39^^,^^45^^,^^48^^,^^52^^,^^54–56^ Spain,^34^^,^^43^^,^^57^ Switzerland,^58^ Italy,^41^^,^^42^^,^^44^ France,^59^ Greece,^49^ Finland^47^ and the UK,^60^ reported having paediatric migrant health screening processes on arrival. However, target populations were not always reached, with a Finnish study reporting one-third of asylum seekers not being reached and some experiencing delays in screening.^47^ This was also noted in a UK study, where a median delay of 6 months between arrival in the UK and infection screening was observed.^60^ A Swiss hospital-based study reported the absence of routine screening procedures in refugee and asylum-seeking inpatient children and the incomplete administration of catch-up immunisations.^58^ Retention in care was also a challenge, with six studies following UAM highlighting a high number of individuals who had tested HBV-positive being lost to follow-up due to rapid relocations,^54^^,^^61^ communication barriers and logistical difficulties in accessing care.^48^^,^^53^^,^^60^^,^^61^

### Pregnant migrants

#### Prevalence

Estimates of CHB prevalence in pregnant migrants reported in 12 studies ranged between 0–7% (Table 2 and Appendix Table A4, pp. 7–8),^38^^,^^62–72^ with five of these studies reporting a significantly higher prevalence of CHB among pregnant migrants compared to native-born individuals.^64^^,^^65^^,^^67^^,^^68^^,^^71^

Undocumented women were also found to have a significantly higher prevalence of Hepatitis B than documented migrants (RR 2.4, 95% C.I. 1.1–5.3)^72^ even after adjustment for region of origin.

Prevalence variation by region of origin was described in an Italian study and found to be highest among pregnant women from the Western Pacific Region, Eastern Europe and Africa (7.0%, 4.0% and 3.3%, respectively).^68^ In this study, more than half (60.6%) of the HBsAg positive pregnant women originated from China and Albania.^68^ Prevalence differences between migrants from specific geographical areas and host populations were examined in two studies.^68^^,^^70^ These established CHB prevalences were significantly higher in pregnant women from China (8.1%), Albania (7.7%), Ukraine (7.2%) and Senegal (6.1%) compared to Italian women (*P* < 0.05)^68^ and in Southeast Asian (primarily Chinese) women (10.8%) compared to native Spanish women (*P* < 0.005).^70^

CHB prevalence estimates by region of origin of pregnant migrants were collated from seven studies. High prevalence estimates (>8%) were found among pregnant migrants from Eastern Europe (*n* = 1 study),^66^ Asia and Pacific (*n* = 1 study)^70^ and Africa (*n* = 1 study).^66^ Intermediate prevalence estimates (2–7%) were found among pregnant migrants from Eastern European (*n* = 4 studies),^68^^,^^70^^,^^72^^,^^73^ Asia and the Pacific (*n* = 3 studies)^68^^,^^72^^,^^73^ and Africa (*n* = 4 studies)^62^^,^^68^^,^^72^^,^^73^ and low prevalence estimates (<2%) were found among pregnant migrants from Eastern Europe (*n* = 2 studies),^62^^,^^72^ Southern Europe (*n* = 1 study)^62^ Asia and the Pacific (*n* = 1 study)^62^ and Latin America and Caribbean (*n* = 1 study)^62^ (Appendix Figure A3, p. 11).

### Disease severity/complications

A fifth of CHB-infected pregnant migrants in one study were HBeAg positive, which is associated with more frequent and rapid progression towards severe liver disease and HCC, and an increased likelihood of vertical transmission.^69^ Most infected pregnant migrants (73.7%) also had detectable HBV DNA, and half had a viral load considered to be associated with chronic liver disease.^69^

### Factors affecting CHB incidence

#### Vaccination status and susceptible population

Only one included study explored vaccination coverage in pregnancy. This study focused on women tested for HBV for the first time in the delivery room in a Greek setting, of whom 70.4% were migrants. Low vaccination-induced protection rates (mean 21.4%) were observed among these women who had missed pre-natal HBV maternal testing, but the results for vaccination coverage were not disaggregated by migrant status.^73^

#### Screening and care pathways

Pregnant migrants (including undocumented migrants) were at significantly greater risk of not being screened for HBV during pregnancy compared to native women across four studies.^68^^,^^72–74^ Foreign citizenship increased the odds of not being screened for HBV by 30% (OR: 1.30, 95% CI: 1.04–1.62) in Italy.^74^ In Greece, among women who had not had prenatal HBV screening, the majority (70.4%) were migrants, and 5.3% of these were subsequently found to have HBV, a significantly higher proportion than in the comparison group (*P* < 0.0001).^73^

Consistent findings were described in a Danish study,^72^ where only 43% of pregnant undocumented migrants had a screening result recorded, compared to 99.9% of pregnant people with legal Danish residency.^72^ Late access to antenatal care was described as an important reason for suboptimal screening coverage by a Danish study, which found that pregnant migrants first accessed antenatal care at a median gestation of 20 weeks (range 6–39 weeks).^63^ A Finnish study also found that 71% of undocumented migrant pregnant women received inadequate prenatal care, with 61% not receiving any antenatal care in the first trimester and 6% receiving no antenatal care at all.^71^ This resulted in missed opportunities to prevent mother-to-child transmission of HBV, as demonstrated by an Irish study of HBV-infected children, where 100% of foreign-born and 30% of Irish-born infected children in the study had not received HBIG or HBV vaccine prophylaxis.^46^ Instances of antiviral treatment not being initiated when indicated and the loss of infected women to follow-up^69^^,^^72^ were also documented.

## Discussion

Our systematic review found intermediate to high CHB prevalence among migrant children in Europe, with a higher prevalence among those originating from the WHO African Region and a higher pooled prevalence among unaccompanied versus accompanied minors. Pooled HBV vaccination coverage estimates were also lower among UAM compared to other child migrants, and a high proportion of migrant children were found to be Hepatitis B non-immune from vaccination or prior infection across multiple geographies of origin, including those from Africa, Latin America, the Balkans, Middle East, Asia and Russia.^34^^,^^41–44^^,^^49^^,^^54^

Our data also show that CHB prevalence was significantly higher among migrant pregnant persons compared to the native-born pregnant population. Undocumented pregnant migrants, those declaring themselves as UAMs (but not recognised as such by the state) and those originating from Eastern Europe, China and Africa were at particularly high risk of CHB.

Based on limited data, our review showed that high HBV viral loads, and complications of CHB, including liver inflammation and fibrosis, are infrequently reported among migrant children.^45–48^ Among pregnant migrants, HBeAg positivity and detectable viral loads were reported among a minority of CHB cases, and the reported experience of antiviral use during pregnancy was limited.^37^

Both migrant children and pregnant persons experience restricted access to healthcare across Europe, leading to a reliance on *ad hoc* care from NGO-run clinics. This may have contributed to the paucity of data on CHB complications and antiviral use in both groups. Children with CHB are typically asymptomatic but may have high viral loads and HBeAg status, meaning they are at risk of transmission to others and also long-term at risk of developing the sequelae of CHB, such as cirrhosis and HCC in adulthood, leading to an increased risk of mortality, which is known to be significantly higher among migrants with CHB compared to native populations in European host countries.^27^ The long-term follow-up and monitoring of young people is therefore vital, to allow for the timely commencement of antiviral therapy coupled with regular age-appropriate counselling about viral transmissibility.^27^^,^^34^ Regular follow-up also provides opportunities to prevent transmission by, e.g. vaccinating close contacts.^75^

Among pregnant migrants, suboptimal antenatal HBV screening due to a lack of access to national pregnancy screening programmes was also reported^72^^,^^73^ with pregnant undocumented migrants having an even poorer chance of being screened than documented migrants, despite having a higher prevalence of HBV.^73^ This could possibly lead to cases of preventable vertical HBV transmission.^71^^,^^73^

The WHO proposes that antenatal care, including equal and timely access to HBV screening, should be easily accessible for all migrants, regardless of legal status and ability to pay.^76^ As part of this, the appropriate administration of antenatal antiviral prophylaxis, plus HBV vaccine and HBIG at birth for the prevention of vertical transmission should be available for all HBV positive migrants. Given the complexities in the supply and availability of these specialist treatments, this needs to be integrated into the regular healthcare system.^77^^,^^78^ However, the reliance on NGOs to fulfil this screening and prevention role for undocumented migrants is a reality in several European countries, leading to frequent loss of follow-up and failure to administer appropriate neonatal prophylaxis to those most at risk.^46^^,^^69^^,^^72^

While specific WHO guidelines exist for the prevention and treatment of CHB in migrant populations through screening, vaccination and equitable access to CHB treatments,^79^^,^^80^ evidence from this review suggests that there is no universal approach to HBV screening in migrant groups across Europe and that policy and practice gaps remain, specifically for children and pregnant migrant groups. Migrants from intermediate- and high-prevalence countries are not always screened,^81^ with some countries relying purely on symptom or patient-initiated testing approaches.^82^ Catch-up immunisation programmes are also not always available in European host countries,^83^^,^^84^ despite data indicating low HBV vaccination coverage in child migrants, below global and regional average rates.

The strengths of this study include the robust systematic methodology employed, including the meta-analyses and pooled estimates obtained. The inclusion of research studies in all languages also enabled us to capture a wide breadth of literature representative of all of Europe.

Limitations of this systematic review include the fact that most studies were cross-sectional and described the experiences of single centres, the majority of which were specialised migrant health settings. Therefore, the study populations may not necessarily have been representative of all migrant types in the reporting country. There was also a lack of consistency in the terminology used to describe different migrant populations. This has implications in the ascertainment of the true ‘at-risk’ population when estimating prevalence. In some studies, prevalence estimates in certain migrant groups may have been underestimated due to a lack of comprehensive screening programmes, language and cultural barriers, as well as stigma and discrimination that might have resulted in high-risk migrants not being captured by the studies.^40^^,^^64^^,^^85^ The calculation of vaccination coverage also varied between studies,^49^^,^^52^ and may have been potentially underestimated in cases where anti-HBS was measured prior to booster administration or in cases of co-infection with HIV (where vaccination may not induce an anti-HBS response) or in rare cases of vaccine non-responders.^52^ It is also worth noting that most studies originated from a small proportion of primarily Western, Northern or Central European countries, and some relied on data over a decade old.^18^^,^^27^^,^^36^^,^^43^^,^^46^^,^^57^^,^^66^^,^^70^^,^^73^^,^^74^ Two studies carried out in similar geographical regions and time periods may have had some overlapping cases.^67^^,^^68^ Caution should therefore be employed when generalising the findings to wider migrant populations.

The findings of this review highlight the need for several policy and clinical recommendations. First and foremost, national and international European policies should incorporate CHB screening and the provision of robust HBV vaccination programmes for migrant children and pregnant persons. Together, but simultaneously, flexible systems are required to enable opportunistic immunisation while still connecting to central reporting systems to enable follow-up data to be collected. This is supported by evidence that high numbers of migrants are HBV non-immune^86^ and that strategies to bolster screening and HBV vaccination efforts in migrant populations in Europe are cost-effective^87^^,^^88^ and reduce resource impact on healthcare systems.^89^

These interventions should be delivered alongside existing preventative health services in maternal and child health so that hepatitis B risk is addressed together with a range of other health inequalities for these populations.^90^ These should include robust childhood and adolescent catch-up vaccination programmes and antenatal infection screening for the prevention of congenital infections, the latter being in line with the WHO’s triple elimination programme, which aims to synergise efforts to eliminate the vertical transmission of HIV, syphilis and HBV. There is a need to capitalise on existing mobile technologies developed during the COVID-19 pandemic to develop digital migrant mobile vaccination and personal health records that are easily accessible to healthcare providers. This would improve the continuum of care for migrants with CHB and improve the efficiency and streamlining of screening procedures.^48^

Future research should endeavour to optimise the description of the origin of migrants, including their migration status, as well as geographical regions of origin, to facilitate evidence synthesis as well as disaggregation of age. Expansion of the research to include migrants to other high-income and low HBV prevalence contexts, such as North America and Australia, would also provide further relevant context and generalisability. Research on post-arrival transmission of CHB, the ongoing health needs of migrant children and young people living with CHB, as well as qualitative research in the field would provide valuable contextual data for quantitative findings.

Large-scale migration from high-prevalence countries has shifted the HBV landscape in Europe, potentially rendering existing elimination strategies inadequate and allowing at-risk migrant groups to go undiagnosed and untreated.^91^ Addressing the CHB health needs of child and pregnant migrants in Europe will require further evidence generation and advocacy in order to design equitable non-hostile health policies that are integrated into broader inclusive social policies that are responsive to the changing epidemiology and migrant profiles.^89^

## Supplementary Material

Appendix_28_03_taae094

## Data Availability

All data is available upon reasonable request to the co-authors.
