# Supplementary material for: Hepatitis B infection and immunity in migrant children and pregnant persons in Europe: a systematic review and meta-analysis
Source: J Travel Med. 2024 Jul 11;31(6):taae094. doi: 10.1093/jtm/taae094 (PMC11298050; doi:10.1093/jtm/taae094)
Supplement: Appendix_28_03_taae094 [file appendix_28_03_taae094.docx]

**Appendix**

**Table 1: Eligibility criteria for evidence selection**

|  | **Inclusion** | **Exclusion** |
| --- | --- | --- |
| **Evidence** | Studies: Observational, experimental, case reports, outbreak reports, systematic reviews  Grey literature | Comments, editorials  Single case studies  Conference/poster abstracts (where no supporting article identified)  Study protocols  Full text unavailable |
| **Population** | Migrant (defined as any individual born outside the country of study) pregnant women and/or children under 18 years.  i.e. first generation migrants | Data not disaggregated by migrant status (or country of origin) and by age and/or pregnancy status  Data only presented on specific subgroups of HBV-infected populations (such as dual Hep B/C or HIV infected, acute hepatitis) |
| **Context** | All migrants in the United Kingdom (UK), EU, European Economic Area (EEA) and Switzerland* | Migrants in other destination countries exclusively or within sample |
| **Content** | CHB prevalence (primary outcome)  Factors affecting CHB incidence (secondary outcome) including screening, transmission, vaccination coverage/susceptibility, healthcare access and linkage to care. | Data on these concepts without any specific reference to HBV.  Modelled data only  Studies dealing with hepatitis other than type B |
| **Date Range** | Published between 1 January 2012 and 8 June 2022 (searches conducted) | Published before 1 January 2012 |

**Full list of countries included below*

## List of destination European countries included

| Austria | Greece | Norway |
| --- | --- | --- |
| Belgium | Hungary | Poland |
| Bulgaria | Iceland | Portugal |
| Cyprus | Ireland | Romania |
| Croatia | Italy | Slovakia |
| Czech Republic | Latvia | Slovenia |
| Denmark | Liechtenstein | Spain |
| Estonia | Lithuania | Sweden |
| Finland | Luxembourg | Switzerland |
| France | Malta | The United Kingdom |
| Germany | The Netherlands |  |

**Box 1. Migrant Definitions**


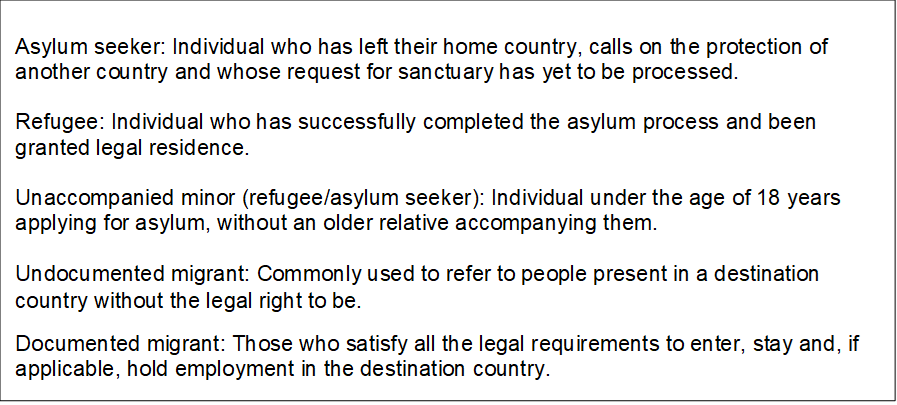


## **Figure 1. Example of detailed search strategy – in EMBASE**

| ‘Migrant’ Terms |  |  |
| --- | --- | --- |
| 1. | migrant*.mp. or exp migrant/ or exp migrant worker/ |  |
| 2. | exp immigration/ or immigrat*.mp. or exp immigrant/ |  |
| 3. | immigration/ or emigration/ or migration/ or emigrat*.mp. |  |
| 4. | exp migration/ or migrati*.mp. |  |
| 5. | exp immigrant/ or exp migrant/ or immigrant*.mp. |  |
| 6. | exp emigrant/ or emigrant*.mp. |  |
| 7. | unaccompanied child.mp. or exp refugee/ |  |
| 8. | exp foreigner/ or foreign born*.mp. |  |
| 9. | foreign-origin.mp. |  |
| 10. | foreign*.mp. |  |
| 11. | exp asylum seeker center/ or exp asylum seeker/ or asylum.mp. |  |
| 12. | exp refugee camp/ or refugee.mp. or exp refugee/ or exp refugee crisis/ |  |
| 13. | non-citizen*.mp. |  |
| 14. | citizenship.mp. or exp citizenship/ |  |
| 15. | nationality*.mp. |  |
| 16. | exp undocumented immigrant/ or undocumented*.mp. |  |
| 17. | illegal*.mp. |  |
| 18. | non-resident*.mp. |  |
| 19. | transient*.mp. |  |
| 20. | expat*.mp. |  |
| 21. | newcomer*.mp. |  |
| 22. | new-comer.mp. |  |
| 23. | 1 or 2 or 3 or 4 or 5 or 6 or 7 or 8 or 9 or 10 or 11 or 12 or 13 or 14 or 15 or 16 or 17 or 18 or 19 or 20 or 21 or 22 |  |
| ‘Prevalence’ Terms |  |  |
| 24. | exp prevalence/ or prevalen*.mp. or exp epidemiology/ |  |
| 25. | exp incidence/ or inciden*.mp. |  |
| 26. | epidemiol*.mp. or exp epidemiology/ |  |
| 27. | exp case study/ or exp case fatality rate/ or exp case control study/ or case*.mp. or exp case report/ |  |
| 28. | outbreak*.mp. |  |
| 29. | exp mortality/ or mortalit*.mp. |  |
| 30. | positivity.mp. or exp positivity rate/ |  |
| 31. | seropositive.mp. |  |
| 32. | positive.mp. |  |
| 33. | exp seroprevalence/ or seroprevalen*.mp. |  |
| 34. | exp case report/ |  |
| 35. | exp epidemic/ |  |
| 36. | exp seroepidemiology/ |  |
| 37. | 24 or 25 or 26 or 27 or 28 or 29 or 30 or 31 or 32 or 33 or 34 or 35 or 36 |  |
| ‘Hepatitis B’ Terms |  |  |
| 38. | exp hepatitis B vaccine/ or exp hepatitis B/ or Hep*B.mp. |  |
| 39. | HBV.mp. or exp Hepatitis B virus/ |  |
| 40. | chronic hepatitis.mp. or exp chronic hepatitis B/ or exp chronic hepatitis/ |  |
| 41. | hbsag.mp. or exp hepatitis B surface antigen/ |  |
| 42. | "hbs ag".mp. or exp hepatitis B antigen/ |  |
| 43. | exp hepatitis B antibody/ or hepatit* b antibody.mp. |  |
| 44. | "hepatitis b surface antigen".mp. |  |
| 45. | 38 or 39 or 40 or 41 or 42 or 43 or 44 |  |
| ‘Europe’ Terms |  |  |
| 46. | (Austria* or Belgium or Belgian or Bulgaria* or Croatia* or Cyprus or Cypriot or Czech* or Denmark or Danish or Estonia* or Finland or Finnish or France or French or German* or Greece or greek or Hungar* or Iceland* or Ireland or Irish or Ital* or Latvia* or Liechtenstein or Lithuania* or Luxembourg* or Malta or Maltese or Netherlands or Dutch or Norway or Norweigan or Poland or Polish or Portug* or Romania* or Slovakia* or Slovenia* or Spain or Spanish or Sweden or Swedish or Switzerland or Swiss or Great Britain or British or United Kingdom or UK or England or English or Wales or Welsh or Scotland or Scottish).ti,ab,kf,sh. |  |
| 47. | (EU or EEA or Europe*).ti,ab,kf,sh. |  |
| 48. | 46 or 47 |  |
| 49. | 23 and 37 and 45 and 48 |  |
| Date Restriction |  |  |
| 50. | limit 49 to yr="2012 -Current" |  |

**Table 2. Mapping of included studies by themes addressed in population, context and concept domain**

| **Theme** | **Paediatric Migrant Studies** | **Pregnant Migrant Studies** | **Total** |
| --- | --- | --- | --- |
| **Concept** | **45** | **22** | **67** |
| Prevalence chronic HBV in migrants | 23 | 12 | 35 |
| Care Pathway | 0 | 5 | 5 |
| Screening Coverage | 1 | 4 | 5 |
| Vaccination Coverage | 13 | 1 | 14 |
| Susceptible Population Proportion | 8 | 0 | 8 |
| **Context** | **27** | **15** | **42** |
| Routine antenatal Care | 0 | 11 | 11 |
| Presentations to Specialised Migrant healthcare facilities | 3 | 2 | 5 |
| On arrival screening | 18 | 1 | 19 |
| Research study | 3 | 1 | 4 |
| Routine healthcare contacts | 3 | 0 | 3 |
| **Population** | **27** | **15** | **42** |
| Migrants (mixed) | 6 | 10 | 16 |
| Undocumented migrants | 0 | 3 | 3 |
| Migrants from Intermediate/high endemicity areas | 1 | 1 | 2 |
| Refugees and/or asylum seekers (may include unaccompanied) | 5 | 1 | 6 |
| Internationally Adopted Children | 2 | 0 | 2 |
| Unaccompanied minors only | 13 | 0 | 13 |

**Table 3. Risk of bias scores for all studies using Newcastle-Ottawa Scale (NOS) for assessing the quality of nonrandomised studies in meta-analysis**

| NOS Criteria: Adapted Cross-Sectional | | | Selection (max 3) | Comparability (max 2) | Exposure (max 3) | Total Score | Risk of Bias* |
| --- | --- | --- | --- | --- | --- | --- | --- |
|  | | | Is the sample representative of the average target population and a satisfactory size? Was comparability between included and non-included subjects satisfactory? | Are subjects in different outcome groups comparable, based on study design or analysis? Are confounding factors controlled? | Were outcome assessments given and were statistical tests described appropriately? |  |  |
| Population | Author | Year |  |  |  |  |  |
| Pregnancy | Santiago | 2012 | 1 | 0 | 3 | 4 | High |
|  | Lopez-Fabal | 2013 | 2 | 1 | 2 | 5 | Medium |
|  | Ruffini | 2014 | 2 | 0 | 3 | 5 | Medium |
|  | Ehmsen | 2014 | 2 | 0 | 2 | 4 | High |
|  | Cochrane | 2015 | 2 | 0 | 3 | 5 | Medium |
|  | Ruffini | 2016 | 2 | 0 | 3 | 5 | Medium |
|  | Sagnelli | 2016 | 2 | 0 | 3 | 5 | Medium |
|  | Wendlend | 2016 | 1 | 0 | 3 | 4 | High |
|  | Lembo | 2017 | 3 | 0 | 3 | 6 | Medium |
|  | Dalmartello | 2019 | 3 | 0 | 3 | 6 | Medium |
|  | Ruiz-Extemera | 2020 | 2 | 0 | 3 | 5 | Medium |
|  | Lo Guidice | 2021 | 2 | 0 | 3 | 5 | Medium |
|  | Tasa | 2021 | 1 | 0 | 3 | 4 | High |
| Population | Author | Year | Selection (max 3) | Comparability (max 2) | Exposure (max 3) | Total Score | Risk of Bias |
| Paediatrics | Hahne | 2012 | 3 | 0 | 3 | 6 | Medium |
|  | Hubschen | 2012 | 2 | 0 | 2 | 4 | High |
|  | Belhassen-Garcia | 2015 | 2 | 0 | 2 | 4 | High |
|  | Finnegan | 2015 | 2 | 0 | 1 | 3 | High |
|  | Jablonka | 2015 | 3 | 0 | 3 | 6 | Medium |
|  | Hampel | 2016 | 2 | 0 | 3 | 5 | Medium |
|  | Marquardt | 2016 | 1 | 0 | 3 | 4 | High |
|  | Mockenhaupt | 2016 | 2 | 0 | 2 | 4 | High |
|  | Monpierre | 2016 | 2 | 0 | 2 | 4 | High |
|  | Pauti | 2016 | 3 | 0 | 3 | 6 | Medium |
|  | Theuring | 2016 | 2 | 0 | 2 | 4 | High |
|  | Maasen | 2017 | 3 | 0 | 2 | 5 | Medium |
|  | Pavlopoulou | 2017 | 2 | 0 | 2 | 4 | High |
|  | Pohl | 2017 | 2 | 0 | 3 | 5 | Medium |
|  | Sollai | 2017 | 3 | 2 | 3 | 8 | Low |
|  | Giordano | 2018 | 0 | 0 | 3 | 3 | High |
|  | Kloning | 2018 | 2 | 0 | 2 | 4 | High |
|  | Tiitala | 2018 | 3 | 0 | 3 | 6 | Medium |
|  | Genton | 2019 | 2 | 0 | 0 | 2 | High |
|  | Hourdet | 2020 | 3 | 0 | 2 | 5 | Medium |
|  | Janda | 2020 | 2 | 0 | 2 | 4 | High |
|  | Marrone | 2020 | 2 | 0 | 3 | 5 | Medium |
|  | Williams | 2020 | 2 | 0 | 1 | 3 | High |
|  | Bergevin | 2021 | 2 | 0 | 3 | 5 | Medium |
|  | Norman | 2021 | 2 | 0 | 2 | 4 | High |
|  | Olivan-Gonzalvo | 2021 | 2 | 0 | 3 | 5 | Medium |
|  | | | | | | | |
| NOS Criteria: Cohort Study | | | Selection (max = 4) | Comparability (max = 2) | Outcome (max = 3) | Total Score | Risk of Bias* |
|  | | | Is the sample representative of the exposed cohort? Is the selection process and ascertainment of exposure adequate? Is there a demonstration that the outcome of interest Is not present at the start of the study? | Are the cohorts comparable on the basis of design or analysis? | Is the assessment of the outcome adequately done?  Is follow-up of acceptable duration and any participants lost to follow up adequately explained? |  |  |
| Population | Author | Year |  |  |  |  |  |
| Pregnancy | Karatapanis | 2012 | 3 | 0 | 3 | 6 | Medium |
| Pregnancy | Dopfer | 2018 | 3 | 1 | 1 | 5 | High |
|  | | | | | | | |
| Paediatrics | Fougere | 2018 | 4 | 0 | 3 | 7 | Medium |

* Quality assessment of all studies was carried out using the Newcastle-Ottawa scoring (NOS) system (score out of 9) and an adapted scoring system for cross-sectional studies (score out of 8). For cross-sectional studies, scores of 0–4 were classified as high risk of bias, scores of 5–6 were classified as moderate risk of bias and scores ≥7 were considered as low risk of bias. For cohort studies, those that scored a total of 8 or 9 points were considered to have low risk of bias; 7 or 6 points were considered to have a medium risk of bias; 5 points or less were considered to have a high risk of bias.

**Table 4. CHB prevalence estimates for child migrants (where reported)**

| **Study/year** | **Destination Country** | **Population** | **Age Range (years)** | **Sample size** | **Prevalence CHB (%)** |
| --- | --- | --- | --- | --- | --- |
| Jablonka, 2017^43^ | Germany | Refugees & asylum seekers | 0-17 | 91 | 0.0% |
| Mockenhaupt, 2016 ^44^ | Germany | UAM (Syrian) | 0-18 | 488 | 0.0% |
| Pavlopoulou, 2017 ^45^ | Greece | Migrant & refugee children | 0-18 | 300 | 0.0% |
| Giordano, 2018^38^ | Italy | IAC | 0-18 | 79 | 0.0% |
| Sollai, 2017 ^39^ | Italy | IAC | 0-18 | 1612 | 0.8% |
| Tiitala, 2018 ^46^ | Finland | Asylum Seekers accompanied and unaccompanied | 0-17 | 9031 | 0.8% |
| Fougere, 2018^47^ | Switzerland | Migrant children | 1-18 | 200 | <1% |
| Hampel, 2016^48^ | Germany | Refugees | 0-17 | 62 | 1.6%  (-) |
| Maasen, 2017 ^48^ | Germany | UAM | 0-18 | 190 | 1.6% |
| Theuring, 2016^36^ | Germany | UAM | 0-18 | 1248 | 1.7% |
| Williams, 2020^49^ | UK | UAM | 0-18 | 211 | 4.8% |
| Marrone, 2020^41^ | Italy | UAM | 0-18 | 879 | 2.5% |
| Olivan-Gonzalvo, 2021^50^ | Spain | UAM (African male) | 0-18 | 622 | 2.6% |
| Genton, 2019^50^ | Switzerland | UAM | 0-18 | 109 | 2.8% |
| Belhassen-Garcia, 2015^40^ | Spain | Migrant children from the WHO African Region North Africa (NA) and Latin America (LA) | 0-18 | 350 | 4.3% |
| Monpierre, 2016^51^ | France | UAM | 0-18 | 235 | 6.0% |
| Janda, 2020^52^ | Germany | UAM | 0-18 | 776 | 7.7% |
| Marquardt, 2016^42^ | Germany | UAM | 12-18 | 101 | 7.9% |
| Kloning, 2018^53^ | Germany | UAM | 0-18 | 113 | 8.0% |
| Bergevin, 2021^54^ | France | UAM | 0-18 | 90 | 8.0% |
| Norman, 2021^37^ | Spain | Migrant children | 0-20 | 96 | 10.4% |
| Hourdet, 2020^37^ | France | Individuals self-reporting as UAM but not state-recognised | 0-18 (self-reported) | 301 | 12.8% |
| Hahne, 2013^16^ | Netherlands | Migrant children | 0-29 | 0-14 yrs: 1476  15-29 yrs: 1002 | 0-14: 2.3%  15-29: 22.3% |

IAC - internationally adopted children; UAM – unaccompanied minor

**Table 5. CHB prevalence estimates for pregnant migrant women (where reported)**

| **Study/year** | **Country** | **Population** | **Sample size*** | **Prevalence in migrant sample (%)** | **Prevalence in native born sample (%) (where measured in the study population)** | **Estimated differences (p-value)** |
| --- | --- | --- | --- | --- | --- | --- |
| Dopfer, 2018^35^ | Germany | Refugee women pregnant on arrival | 9 | 0.00% | N/A | N/A |
| Ehmsen, 2014^63^ | Denmark | Pregnant undocumented migrant women | 96 | 1.00% | N/A | N/A |
| Lopez-Fabal, 2013^68^ | Spain | Pregnant women | 2,752 migrants (8,012 total) | 1.65% | 0.40% | No data |
| Cochrane, 2015^62^ | UK | Pregnant migrant women from regions with HBV prevalence >2% | 5840 | 1.70% (95% C.I. 1.4–2.1) | N/A | N/A |
| Santiago, 2012^70^ | Spain | Pregnant women | 1,214 migrants (91 Spanish) | 2% | 1.10% | No data |
| Lo Giudice, 2021^65^ | Italy | Pregnant women | 727 migrants (6,896 total) | 2.10% | 0.20% | P<0.0001  [chi-square significance testing] |
| Ruffini, 2016^69^ | Italy | Pregnant women | 2563 migrants (10232 total) | 2.70% | 0.20% | p<0.05 |
| Lembo, 2017^64^ | Italy | Pregnant women | 711 migrants (6128 total) | 3.00% | 0.20% | p<0.001  [2-sided significance testing] |
| Tasa, 2021^71^ | Finland | Pregnant undocumented migrant women | 51,447 total (62 undocumented) | 3.40% | 0.20% | p<0.007 [calculated using Fisher’s Exact Test] |
| Ruffini, 2014^67^ | Italy | Pregnant women | 397 migrants (1651 total) | 4.30% | 0.40% | P<0.001 |
| Wendland, 2016^72^ | Denmark | Pregnant undocumented migrant women | 94 | 6.10% | N/A | N/A |
| Sagnelli, 2016^69^ | Italy | Pregnant migrant women | 1970 | 7.26% | N/A | N/A |

**quoted for prevalence estimate in pregnant women – may differ from total study sample*

**Figure 5. Child migrant percentage vaccination coverage and susceptible population estimates from studies reporting data.** *Abbreviations in figure: IAC: internationally adopted children, SSA: Sub-Saharan Africa, NA: North Africa, LA: Latin America; UAM: Unaccompanied migrants*

**Table 5: Hepatitis B Vaccination Coverage (%) by WHO Region 2012 – 2020 for HepB birth dose* and HepB3** Data Source: WHO ^8^**

| **Vaccination Indicator by Region** | **Year** | | | | | | | | |
| --- | --- | --- | --- | --- | --- | --- | --- | --- | --- |
|  | **2012** | **2013** | **2014** | **2015** | **2016** | **2017** | **2018** | **2019** | **2020** |
| Africa | | | | | | | | | |
| HepB, birth dose | 3 | 3 | 3 | 3 | 4 | 4 | 4 | 4 | 6 |
| HepB3 | 70 | 69 | 70 | 70 | 72 | 73 | 73 | 73 | 72 |
| Eastern Mediterranean | | | | | | | | | |
| HepB, birth dose | 18 | 19 | 19 | 19 | 20 | 33 | 34 | 35 | 35 |
| HepB3 | 76 | 79 | 79 | 80 | 81 | 83 | 84 | 85 | 81 |
| Europe | | | | | | | | | |
| HepB, birth dose | 40 | 41 | 41 | 41 | 40 | 39 | 40 | 41 | 41 |
| HepB3 | 81 | 82 | 81 | 82 | 82 | 84 | 84 | 92 | 91 |
| The Americas | | | | | | | | | |
| HepB, birth dose | 52 | 54 | 53 | 54 | 49 | 53 | 55 | 54 | 60 |
| HepB3 | 91 | 89 | 89 | 89 | 90 | 83 | 83 | 79 | 81 |
| South-East Asia | | | | | | | | | |
| HepB, birth dose | 17 | 27 | 29 | 31 | 34 | 45 | 49 | 54 | 51 |
| HepB3 | 78 | 77 | 83 | 88 | 89 | 90 | 90 | 91 | 85 |
| Western Pacific | | | | | | | | | |
| HepB, birth dose | 80 | 82 | 81 | 84 | 84 | 85 | 84 | 85 | 84 |
| HepB3 | 93 | 90 | 90 | 90 | 93 | 92 | 91 | 95 | 95 |
|  |  |  |  |  |  |  |  |  |  |

**HepB, birth dose: percentage in target population who received HBV vaccine dose within first 24 hours of birth in given year*

***HepB3: percentage in target population who received three doses of Hepatitis B containing vaccine in given year*

**Figure 6. Number of studies reporting CHB prevalence estimates among pregnant migrants by region of origin and prevalence category**.^71^


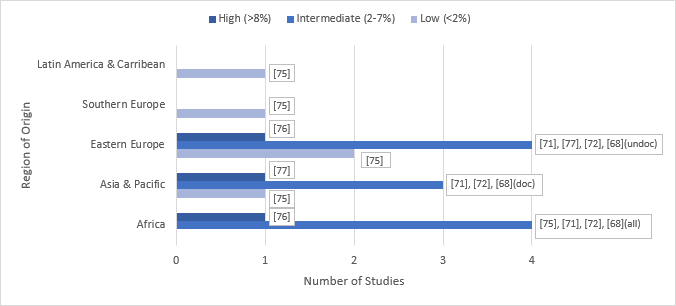


*doc = prevalence reported for documented migrants, **undoc = prevalence reported for undocumented migrants
